# NOX Activation by Subunit Interaction and Underlying Mechanisms in Disease

**DOI:** 10.3389/fncel.2016.00301

**Published:** 2017-01-10

**Authors:** Radhika Rastogi, Xiaokun Geng, Fengwu Li, Yuchuan Ding

**Affiliations:** ^1^Department of Neurosurgery, Wayne State University School of MedicineDetroit, MI, USA; ^2^China-America Institute of Neuroscience, Beijing Luhe Hospital, Capital Medical UniversityBeijing, China; ^3^Department of Neurology, Beijing Luhe Hospital, Capital Medical UniversityBeijing, China

**Keywords:** NAPDH oxidase, NOX inhibitors, reactive oxygen species, stroke, ischemia/reperfusion, neurodegenerative disease, TBI, PKC

## Abstract

Nicotinamide adenine dinucleotide phosphate (NAPDH) oxidase (NOX) is an enzyme complex with the sole function of producing superoxide anion and reactive oxygen species (ROS) at the expense of NADPH. Vital to the immune system as well as cellular signaling, NOX is also involved in the pathologies of a wide variety of disease states. Particularly, it is an integral player in many neurological diseases, including stroke, TBI, and neurodegenerative diseases. Pathologically, NOX produces an excessive amount of ROS that exceed the body’s antioxidant ability to neutralize them, leading to oxidative stress and aberrant signaling. This prevalence makes it an attractive therapeutic target and as such, NOX inhibitors have been studied and developed to counter NOX’s deleterious effects. However, recent studies of NOX have created a better understanding of the NOX complex. Comprised of independent cytosolic subunits, p47-*phox*, p67-*phox*, p40-*phox* and *Rac*, and membrane subunits, gp91-*phox* and p22-*phox*, the NOX complex requires a unique activation process through subunit interaction. Of these subunits, p47-*phox* plays the most important role in activation, binding and translocating the cytosolic subunits to the membrane and anchoring to p22-*phox* to organize the complex for NOX activation and function. Moreover, these interactions, particularly that between p47-*phox* and p22-*phox*, are dependent on phosphorylation initiated by upstream processes involving protein kinase C (PKC). This review will look at these interactions between subunits and with PKC. It will focus on the interaction involving p47-*phox* with p22-*phox*, key in bringing the cytosolic subunits to the membrane. Furthermore, the implication of these interactions as a target for NOX inhibitors such as apocynin will be discussed as a potential avenue for further investigation, in order to develop more specific NOX inhibitors based on the inhibition of NOX assembly and activation.

## Introduction

Reactive oxygen species (ROS) have been identified as essential players in an increasing number of disease states, exacerbating oxidative stress and facilitating tissue dysfunction. However, the origin of ROS varies widely. They originate from both exogenous sources, such as radiation or drugs, and endogenous sources, including mitochondria, peroxisomes, and a host of enzymes which include xanthine oxidase, nitric oxide synthetase, p450 cytochrome, and most importantly, nicotinamide adenine dinucleotide phosphate (NADPH) oxidase (NOX) (Brieger et al., 2012). Of these, NOX is the only one with the exclusive role in producing ROS and is the fundamental source of ROS in our body.

NOX is a family of integral enzyme complexes present prevalently throughout the body. Classically known for its role in phagocytic leukocytes, NOX generates superoxide anion (O_2_•–) from molecular oxygen at the expense of NADPH when activated in these cells in order to destroy phagocytosed organisms and facilitate their anti-microbial function. However, its presence is not limited to leukocytes but has also been found in most tissues of the body. Its production of ROS has also been implicated in biosignaling and cell function as well as in apoptotic regulation (Sumimoto et al., 2005; D’Autréaux and Toledano, 2007). To prevent ROS overproduction, NOX activation is heavily modulated through its activation via phosphorylation. Such phosphorylation is facilitated by stimuli such as activation via phagocytic particles or by physiological or pathological cues, particularly cellular stresses (Jiang et al., 2011). These cues include altered nutritional status, such as hyperglycemia which can lead to protein kinase C (PKC)-activated NOX activation, altered cellular environments, such as hypoxia, and altered chemical environments due to inflammatory and stressed states (Jiang et al., 2011; Shao and Bayraktutan, 2014).

Beyond its role in defense and signaling, NOX is a primary player in oxidative stress, the condition where ROS levels exceed the body’s antioxidant defense mechanisms. With uncompensated increases in ROS, increases in lipid peroxidation, cell death, matrix metalloprotease (MMP) production, DNA damage and deleterious signaling cascades ensue. Such pathologic production of ROS can lead to a host of disease states. Excessive NOX production of ROS as well as upregulation of NOX is seen in numerous disease states, including those involving inflammation, diabetes, and cancer (Tang et al., 2012). Moreover, it plays a key role in neurological pathologies. As a main producer of ROS within the central nervous system (CNS), NOX contributes greatly to subsequent brain damage resulting from traumatic brain injury, ischemia/reperfusion injury in stroke, and to the pathologies of neurodegenerative diseases (Tang et al., 2011). As a result of its pervasive action, NOX has become an attractive target for therapies and drugs, but understanding its assembly and activation process is critical to developing therapeutic systems with appropriate specificity.

## NOX Structure, Subunit Interactions, and Activation

### Structure and Subunits

**Figure 1:**
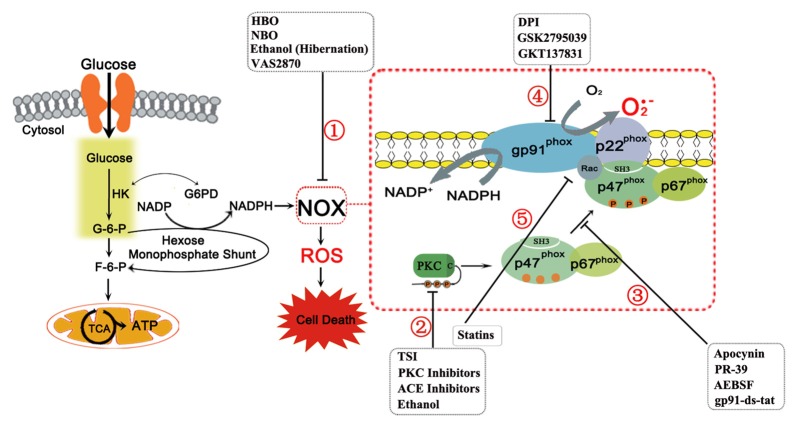
**The nicotinamide adenine dinucleotide phosphate (NAPDH) oxidase (NOX) complex and its subunits are shown above with details pertaining to its activation sequence**. The NOX complex consists of two membrane subunits (gp91-*phox*, or its homologs, and p22-*phox*) that form the catalytic core of NOX, several cytosolic subunits (p47-*phox*, p67-*phox*, p40-*phox* (not shown)), and the G-protein* Rac*, which are required for assembly and activation. In NOX activation, stimuli induce Protein Kinase C (PKC) activation, which phosphorylates p47-*phox*, already complexed to p67-*phox*, and reveals the p47-*phox* SH3 domain. The cytosolic subunits then translocate to the membrane due to interactions between the SH3 domains of p47-*phox* with the proline rich region of p22-*phox*. *Rac* independently translocates to the complex to activate NOX. When activated, NOX produces superoxide ion through a redox reaction with molecular oxygen and NADPH. The latter is produced from glucose, which enters the cell and as an intermediate of glycolysis, produces glucose-6-phosphate (G-6-P). This substrate may continue through glycolysis or may be shunted to the hexose monophosphate shunt to produce NADPH by reducing NADP^+^. The remaining carbon backbone is shunted back to the glycolytic process downstream of G-6-P to fructose-6-phosphate (F-6-P) and then enters the tricarboxylic acid (TCA) cycle and electron transport chain to produce energy as adenosine triphosphate (ATP) in mitochondria. However, the NADPH produced, with molecular oxygen, acts as a substrate for NOX to produce reactive oxygen species (ROS). In disease states, ROS overproduction leads to cell death. NOX inhibitors targeted to prevent this are also shown with their location of action. NOX inhibition can function through several pathways: (1) By acting on NOX via an unspecified mechanism; (2) By acting on the PKC isoforms or upstream to PKC to prevent NOX activation by inhibiting phosphorylation; (3) By inhibiting the interactions of p47-*phox* with NOX subunits, particularly p22-*phox*, preventing NOX activation by inhibiting assembly; (4) By acting directly on gp91-*phox* or its homologs, preventing NOX catalytic activity; and (5) By preventing *Rac* translocation to the NOX complex to prevent NOX activation.

With its prevalence in disease, it is important to characterize NOX in its structure. The classical NOX found in neutrophils is an enzyme complex that is comprised of several subunits, including two membrane subunits (gp91-*phox* (NOX2) and p22-*phox*), three cytosolic subunits (p47-*phox*, p67-*phox*, p40-*phox),* and the G-protein *Rac* (Figure 1). More recently, several NOX complexes have been found with homologs of the gp91-*phox* (NOX2) subunit, which are NOX1, NOX3 to NOX5, DUOX1, and DUOX2. The enzyme complexes take the name of their catalytic homolog. These alternative subunits have unique roles in their respective NOX complexes and will be discussed below.

The NOX complex itself is split between the membrane compartment and the cytosolic compartment at rest. The membrane compartment forms the catalytic core of NOX, the flavocytochrome* b*_558._ In the classical NOX complex, this is comprised of the gp91-*phox* subunit (NOX2) and p22-*phox*. The gp91-*phox* subunit is the main catalytic subunit that transfers NADPH electrons via FAD and heme to molecular oxygen through coupled redox reactions, producing superoxide anion. It constitutively forms a heterodimer with p22-*phox* on the membrane (Yu et al., 1998). However, activation is dependent on the translocation of the cytosolic subunits to the membrane subunits as well as independent activation of *Rac* to fully assemble the complex. The cytosolic subunits depend on phosphorylation for activation. At rest, p40-*phox* and p67-*phox* subunits are commonly complexed in the cytosol and may be associated with p47-*phox* as well. Phosphorylation activates p47-*phox* and unmasks a region to allow it to definitely bind p67-*phox* and form a trimeric cytosolic complex (Tsunawaki and Yoshikawa, 2000; Lapouge et al., 2002). Subsequently, p47-*phox* facilitates their translocation to the membrane, binding primarily to p22-*phox* and assembling the active NOX complex (Ago et al., 2003). Though a complex process, this unique activation process allows for specific modulation at many levels of the NOX complex both prior to activation and in the active state (Groemping and Rittinger, 2005; Sumimoto et al., 2005).

With seven different isoforms, NOX may contain homologs instead of the gp91-*phox* (NOX2) subunit. As the catalytic core of NOX, catalytic function is preserved through structural homology within these homologs NOX1, NOX 3–5, DUOX1, and DUOX2. All have six or seven transmembrane domains, with two heme binding regions containing histidine residues and a NADPH binding region on the intracellular C-terminus to facilitate superoxide production. However, regulation, localization, and function differ slightly across the isoforms. NOX1 is commonly found in the colon, vascular smooth muscle cells, and the stomach (Sumimoto et al., 2005). It is constitutively active due to the subunits with which it interacts. It is found with the homologs NOXO1 and NOXA1 rather than p47-*phox* and p67-*phox*, respectively, which contribute to its constitutive activity, as unlike p47-*phox*, NOXO1 does not need phosphorylation to translocate to and activate NOX1 (Takeya et al., 2003; Sumimoto et al., 2005). Aside from phagocytic cells, including microglia, NOX2 is also found in endothelial cells and neurons. NOX3 is mainly in fetal tissues of the kidney and liver. It is also implicated in the development of otoconia in the inner ear of mice and is localized to adult cochlear and vestibular systems of the inner ear (Bánfi et al., 2004). Its presence in the human ear has yet to be established. NOX4 is localized to the kidneys, cardiovascular endothelial cells and many other cells, as it is also constitutively active. It does not need cytosolic subunits for activation, though they are able to modulate NOX4 activity. It also produces hydrogen peroxide rather than superoxide anion as its product. NOX1–4 depend on complexing with the p22-*phox* subunit and NOX1–3 depend on cytosolic subunits to function. NOX5, found in lymphocytes of the spleen and lymph nodes and in the testes, does not need p22-*phox* to function and has a calcium-dependent activation mechanism facilitated via additional EF-hand motifs in its structure. DUOX1 and 2, dual oxidases, similarly have EF-hand motifs and subsequently, calcium-dependent activation. However, they also have a peroxidase-like ectodomain and are mainly localized to thyrocytes (Sumimoto et al., 2005). With such variety in structure, regulation, activation and localization, the different NOX complex isoforms have differing roles across disease states. Interestingly, however, the NOX2 isoform has been greatly implicated in most diseases due to its association with inflammatory cells migrating to disease sites.

### p47-*phox* and p22-*phox*

Of the NOX subunits, p47-*phox* plays a central role as the organizer and translocator of the subunits for activation. It targets the cytosolic subunits to the membrane via association with p22-*phox*. In several NOX isoforms, it is integral in facilitating NOX activation and function. While the other components (p67-*phox*, p40-*phox*, gp91-*phox*, and* Rac)* are also important in NOX function, their role in NOX assembly and activation is limited, and thus, will not be elucidated further in this review. The function of p47-*phox* is enabled by its structure, with two adjacent *src*-homology 3 (SH3) domains, a polybasic region c-terminal to them, a proline rich region, and a PX (phox) domain for interactions. The proline rich region binds p67-*phox* while the SH3 domains of p47-*phox* interact with proline rich regions of p22-*phox* to create the complex (DeLeo et al., 1995; Huang and Kleinberg, 1999). However, in the resting state, p47-*phox* SH3 domains are hidden due to internal interactions with a polybasic region C-terminal to the SH3 domains, stretching from residues 286 to 340 (Leusen et al., 1996; Ago et al., 1999; Huang and Kleinberg, 1999). This internal interaction basally inhibits NOX activation by preventing the translocation of the cytosolic subunits to the flavocytochrome b_558_ (Ago et al., 1999). The autoinhibited region is unmasked through phosphorylation of several key residues in the C-terminus region from Ser303 to Ser379, inducing a conformational change that disrupts the SH3 domain and C-terminus internal binding and allowing for translocation and binding to the phosphorylated p22-*phox* (Faust et al., 1995; Leusen et al., 1996; Ago et al., 1999; Huang and Kleinberg, 1999; Sumimoto, 2008). Its structure forms a highly regulated and interactive subunit, reflecting its role as the main organizer of NOX.

The p22-*phox* subunit, the target subunit of p47-*phox*, has 195 amino acids with hydrophobic helices in its N-terminus anchoring it to the membrane. It is constitutively associated with gp91-*phox* or its analog in NOX1 to NOX4 to anchor the membrane subunits. Structurally, it contains a proline rich region in its C-terminal tail stretching from residues 149 to 162 that binds to the SH3 domains on the p47-*phox* subunit to allow for the necessary interactions for NOX activation (Leusen et al., 1996). The interaction between the two subunits will be further elucidated below.

### NOX Activation Through Phosphorylation by Protein Kinase C (PKC)

Phosphorylation is a vital part of NOX activation in all NOX isoforms at various stages of activation, even in constitutively active forms. However, subunit phosphorylation is necessary for activation in NOX1 to NOX3. In those isoforms, phosphorylation of p47-*phox* is of particular interest in order to uncover its SH3 domains and facilitate translocation of the cytosolic subunits for activation. However, the mechanism of phosphorylation provides potential for further therapeutic targets and elucidates mechanisms through which disease states potentially progress. Phosphorylation of p47-*phox* may occur through several pathways involving serine kinases, including through PKC isoforms (δ, β, α, ζ), mitogen-activated protein kinases (MAPK), cAMP dependent kinases, p21-activated kinases (PAK), and PKB/AKT (Leusen et al., 1996; Groemping and Rittinger, 2005). However, a primary pathway implicates PKC isoforms in its phosphorylation and subsequent activation, particularly in situations of cellular stress and phagocytic NOX activation (Bey et al., 2004; Jiang et al., 2011). The p47-*phox* subunit is a particular target of PKC and has been found to be phosphorylated with pure PKC lysate but not in mutated versions from patients with autosomal chronic granulomatous disease (CGD) (Kramer et al., 1988). PMA (phorbol 12-myristate 13-acetate), a potent PKC activator, has been shown to induce a respiratory burst of ROS generation by NOX through the stimulation of PKC (Cox et al., 1985). This behavior is the foundation for subsequent studies of NOX activation as well. Furthermore, inhibiting PKC activation, both broadly with staurosporine in PMA-stimulated cells and specifically with PKC-δ inhibitors, prevented NOX activation and ROS generation (Nauseef et al., 1991; Bey et al., 2004).

As most NOX subunits require phosphorylation to become activated and complex together, the targeting of PKC to the p47-*phox* subunit has been demonstrated as well. The key serine residues for activation on p47-*phox*, Ser303, 304 and 328, are residues that are specifically phosphorylated by PKC isoforms. Staurosporine inhibition of PKC resulted in p47-*phox* and p67-*phox* subunits localized exclusively in the cytosol and also attenuated p47-*phox* phosphorylation (Nauseef et al., 1991). PKC-β and PKC-δ isoforms were specifically implicated in p47-*phox* phosphorylation in neutrophils, as both were co-immunoprecipitated with p47-*phox* in PMA-treated cells (Reeves et al., 1999). Moreover, while PKC-δ inhibition prevented p47-*phox* phosphorylation and translocation in monocytes *in vitro*, PKC-α and PKC-ε inhibition had no effect, indicating PKC specificity with activation (Bey et al., 2004). PKC, particularly the α and δ isoforms, was found to additionally phosphorylate the p22-*phox* subunit at its Thr147 position to further promote interaction with p47-*phox* (Lewis et al., 2010). Thus, PKC plays a critical role in activation.

PKC’s role in NOX activation extends to upstream stimuli and pathways that activate PKC itself. Hyperglycemia-stimulated PKC expression affected NOX production of ROS in the blood brain barrier. PKC-α and –β isoforms were elevated and resulted in increased p47-*phox* phosphorylation and NOX activation under hyperglycemic conditions with brain microvascular endothelial cells, astrocytes, and pericytes simulating the blood brain barrier. Inhibition of the PKC isoforms reduced p47-*phox* phosphorylation accordingly (Shao and Bayraktutan, 2013). In hyperglycemic stimulation of PKC, particularly PKC-β_1_, NOX activation was found to potentiate blood brain barrier breakdown and apoptosis. Such states are common in stroke injury (Shao and Bayraktutan, 2013, 2014).

Other upstream pathways activating PKC and subsequently NOX include those of diabetic nephropathy, where advanced glycation end products (AGEs) or advanced oxidation protein products (AOPPs) increased PKC activity, particularly of PKC-α, with resulting increases in NOX (Williams, 2007; Thallas-Bonke et al., 2008; Wei et al., 2009). The PKC-α inhibitor Ro-32-0432 reduced superoxide production and NOX activation, demonstrating PKC’s role in activating NOX (Thallas-Bonke et al., 2008; Wei et al., 2009). Moreover, the angiotensin II (ANGII) axis of ROS production via endothelial NOX similarly implicates PKC. ANGII significantly increased p47-*phox* phosphorylation, increased p47-*phox* interaction with p22-*phox*, and subsequent increased superoxide production (Landmesser et al., 2002; Li and Shah, 2003). In vascular smooth muscle cells, ANGII increased PKC-δ association with p47-*phox* and p47-*phox* translocation to the membrane, inducing ROS production via an ANGII/PKC-δ/p47-*phox* pathway of NOX activation (Lv et al., 2012).

Thus, PKC plays an essential role in activating NOX and PKC itself is activated by several upstream stimuli to facilitate NOX activation. Interestingly, different PKC isoforms act as the main NOX activator in different tissues. PKC-δ is the dominant isoform in leukocytes, associated particularly with NOX2. PKC-β also played a role, but PKC-α did not act in leukocytes (Reeves et al., 1999; Bey et al., 2004). However, PKC-α appears to have a large role in NOX activation in the kidney, where NOX2 and NOX4 dominate. Alternate NOX isoforms may account for the difference in activating PKC isoforms across studies, though definitive preference or association between PKC and NOX isoforms is not yet clear. Moreover, different instigating stimuli, such as hyperglycemia, AGEs, and ANGII, may also affect different PKC isoforms and cause the subsequently observed differences in NOX activation across different PKC isoforms. Elucidating which isoforms are dominant in neurological conditions requires further study, though the inflammatory pathology behind many diseases suggests a larger role of PKC δ and PKC-β.

Lastly, arachidonic acid was found to act synergistically with PKC activation of NOX to increase activation, especially by potentiating the p47-*phox* binding to p22-*phox*. It likely functions through disrupting the p47-*phox* SH3 masking by the polybasic C-terminal region in conjunction with phosphorylation of Ser residues by PKC (Shiose and Sumimoto, 2000). Overall, PKC plays a crucial role in phosphorylation of NOX subunits. This suggests that there is therapeutic potential in manipulating NOX activation through modulating upstream PKC pathways in pathologic states. However, to achieve this therapeutic goal, more specificity regarding the action of specific PKC isoforms and how they affect specific NOX isoforms must be determined.

### Interactions of p47-*phox* and p22-*phox*

NOX activation is heavily modulated, especially through protein-protein interactions. The cytosolic subunits complex through such interactions and subsequently translocate to the membrane subunits to activate the catalytic core of gp91-*phox* (Sumimoto, 2008). One key protein-protein interaction is the binding of p47-*phox* to p22-*phox* during translocation, to anchor the complex of cytosolic subunits to the membrane and provide the correct enzyme configuration for activation (Sumimoto, 2008). As mentioned, this is facilitated by the interaction between the SH3 domains of p47-*phox* and the proline rich regions of p22-*phox*. Unmasking the SH3 domain is necessary to this interaction and is facilitated by phosphorylation, most commonly through the PKC isoforms as previously explained (Groemping and Rittinger, 2005). In the activated NOX complex, the p47-*phox* subunit is the most phosphorylated subunit. Thus far, 11 phosphorylation sites have been identified: serine residues 303, 304, 310, 315, 320, 328, 345, 348, 359, 370, and 379 (Faust et al., 1995).

Only the most phosphorylated p47-*phox* subunits translocate to the membrane, but the residues themselves have been found to play different roles in the process. An initial study found that independent phosphorylation of Ser379 is necessary for p47-*phox* translocation (Faust et al., 1995). It was also found that the residues at Ser359 and Ser370 were necessary in facilitating phosphorylation of subsequent residues, allowing translocation and enabling oxidase activity (Johnson et al., 1998). However, these three residues (Ser379, Ser359 and Ser370) are not sufficient to interact with the p22-*phox* subunit. These residues are also located outside the region interacting with the SH3 domains, from 286 to 340 (Ago et al., 1999). Thus, the Ser359 and Ser370 residues are likely involved in separate aspects of translocation and NOX activation, likely supporting superoxide production rather than unmasking the SH3 domain (Johnson et al., 1998; Ago et al., 1999). Instead, simultaneous phosphorylation of only Ser303, Ser304, and Ser328 was found to be sufficient in inducing the conformational change needed to reveal the SH3 domains for p22-*phox* (Ago et al., 1999). As a result, Ser303, Ser304, and Ser328 are the key serines on p47-*phox* that facilitate targeting of the cytosolic subunits to the membrane at p22-*phox* for proper NOX assembly.

NOXO1 (NOX organizer 1) further demonstrates the functionality of the p47-*phox* subunit. It encodes for a homolog of p47-*phox* and conserves the main interactive domains of p47-*phox* but lacks the polybasic region that masks the SH3 domains in p47-*phox*. Thus, it is constitutively active and able to bind p22-*phox* without phosphorylation. Moreover, superoxide production in cells transfected with NOXO1 was elevated compared to those with p47-*phox* in the absence of any stimulants, demonstrating that the unmasking of SH3 domains is key in organizing and activating NOX. Moreover, this explains the constitutive activity of NOX1, as it is commonly found with NOXO1 (Takeya et al., 2003).

Once exposed, the SH3 domain binds to the proline rich region of p22-*phox*, which anchors the cytosolic p47-p67-p40 complex to the membrane complex for activation (Leusen et al., 1996). Moreover, phosphorylation of the p22-*phox* subunit at the Thr147 position is also integral to the p47-*phox*/p22-*phox* interaction. When replaced with alanine, NOX activity was reduced significantly and phosphorylation of p22-*phox* by the PKC-α and –δ isoforms was prevented altogether (Lewis et al., 2010). Thus, this interaction is key to the recruitment of cytosolic subunits and NOX function.

The importance of the proline rich region in p47-*phox* and p22-*phox* interaction has been demonstrated clinically in a patient with CGD who had a single mutation of proline to glutamine at residue 156 of p22-*phox*. p47-*phox* translocation and association with the membrane was nearly absent in cells stimulated with PMA. The recruitment of p67-*phox* was similarly absent in this patient’s cells without p47-*phox* to organize it. Dysfunction of NOX in the patient resulted from the lack of activation as the catalytic b_558_ unit functioned normally (Leusen et al., 1994a).

For further membrane stabilization, p47-*phox* also has a PX (phox) domain which binds phosphoinositides in the membrane. This domain is similarly masked by interactions with the SH3 domains internal to p47-*phox* and unmasked by phosphorylation, crucial for targeting to the membrane (Ago et al., 2003). p47-*phox* also has multiple binding sites on the gp91-*phox* subunit for further NOX complex stabilization, located along the C-terminal tail of gp91-*phox* as well as on sites more N terminal in the peptide. The region around 500 of gp91-*phox* is clinically critical. A patient suffering from CGD had an Asp to Gly mutation at residue 500 and subsequent p47-*phox* and p67-*phox* recruitment to the membrane fraction was strongly disrupted due to disruption of p47-*phox* binding at the gp91-*phox* region (Leusen et al., 1994b). Thus, these binding sites play additional supplementary roles in p47-*phox* translocation to the flavocytochrome b_558_.

Once bound, the p47-*phox* subunit permits for electron transfers to proceed from the FAD to the heme groups in flavocytochrome b_558_ in order to reach oxygen (DeLeo et al., 1995). The initial transfer from NADPH to FAD only requires the p67-*phox* isoform, the activator subunit (Leusen et al., 1996).

## NOX in Disease

An attractive target for therapeutic inhibition, NOX has been greatly implicated in varied disease processes. Interestingly, it has been upregulated and/or activated in several pathological states such as hyperglycemia, inflammation, cancer, and vascular cases. This review will focus on its role in neurological diseases. A summary of these effects are provided in Table 1.

**Table 1:** **This table summarizes the involvement of nicotinamide adenine dinucleotide phosphate (NAPDH) oxidase (NOX) in the various disease states discussed in the article and the pathologic effect mediated by NOX**.

Disease	NOX involvement	Pathologic effect	Reference
Alzheimer’s Disease	Beta amyloid-induced activation of NOX ROS production NMDA excitotoxicity mediated via NOX ROS Primarily NOX2 studied	Neuronal cell damage/death, secondary to amyloid plaques Cerebral dysfunction and behavior deficits	Bianca et al. (1999); Shimohama et al. (2000); Abramov et al. (2004); Qin et al. (2006); Xia et al. (2007); Park et al. (2008); Ushio-Fukai and Nakamura (2008); Brennan et al. (2009); Kamata (2009); Sorce and Krause (2009); Costa et al. (2014) and Yan et al. (2015)
Parkinson’s Disease	Microglial NOX ROS production and cell damage Alpha-synuclein mediated NOX activation NMDA excitotoxicity (mediated via NOX ROS) ANGII mediated NOX4 activation NOX1, NOX2, and NOX4 identified but without distinguishing priority	Dopaminergic neuronal cell loss Alpha-synuclein aggregation Motor abnormalities Microglial activation	Gandhi et al. (2009); Hirsch and Hunot (2009); Sorce and Krause (2009); Zawada et al. (2011); Choi et al. (2012); Zawada et al. (2015); Sharma and Nehru (2016) and Sharma et al. (2016)
TBI	NOX-induced ROS overproduction NMDA excitotoxicity (mediated via NOX ROS) NOX2 > NOX4	Oxidative stress and secondary brain damage Neuroinflammation	Dohi et al. (2010); Cooney et al. (2013); Lu et al. (2014) and Niesman et al. (2014)
Ischemia	Microglial NOX activation secondary to inflammation/cytokines Hyperglycemia/PKC pathway of NOX activation and MMP induction NOX2 > NOX4 > other NOX isoforms Hyperglycemia/PKC/NOX activation	ROS mediated direct cell/membrane damage Blood Brain Barrier breakdown: Endothelial apoptosis MMP-induced tight junction dysfunction Increased reperfusion injury and infarct size Increased hemorrhage with t-PA reperfusion	Kleinschnitz et al. (2010); Chen et al. (2011); Tang et al. (2011); Won et al. (2011); Woodfin et al. (2011); Tang et al. (2012); Kochanski et al. (2013); Shao and Bayraktutan (2013, 2014) and Shen et al. (2016)
ALS	NOX-induced ROS overproduction and protein oxidation Mutant SOD1 binding *Rac* to active form, increasing NOX activity Primarily NOX2 studied	Direct ROS mediated damage and oxidative stress Modified IGF1/AKT survival pathways, causing motor neuron death	Simpson et al. (2003); Wu et al. (2006); Boillée and Cleveland (2008) and Harraz et al. (2008)

*NOX involvement covers the relevant isoforms, if specified, as well as the pathway through which it acts to cause damage. Pathologic effect specifies the resulting pathology due to NOX invovlement in the disease state*.

### Neurodegenerative Diseases

A particular field of interest is targeting NOX in the CNS as it is implicated in numerous diseases of the CNS and acts as its main contributor of ROS. Through its inflammatory actions and oxidative stress, it is also involved in neurodegenerative diseases such as Alzheimer’s disease (AD) and Parkinson’s Disease (PD). Moreover, NMDA receptor activation leads to superoxide production, primarily via NOX. Thus, sustained NMDA activation can lead to NMDA excitotoxicity, mediated by NOX-induced ROS overproduction. As a core mechanism of several neurodegenerative diseases as well as stroke, this aspect adds to the potential protection of NOX inhibition (Brennan et al., 2009).

With differing isoforms present in all neuronal and glial cells, NOX plays a role in exacerbating AD presentation by worsening the effects of neurofibrillary tangles and β-amyloid plaques, though they develop without NOX involvement (Sorce and Krause, 2009). Beta-amyloid plaques have been found to activate NOX in astrocytes and microglia, with the cytosolic p47-*phox* fraction migrating to the membrane (Bianca et al., 1999; Shimohama et al., 2000; Abramov et al., 2004; Qin et al., 2006). NOX inhibition of a neuron-astrocyte culture and a neuron-microglia culture was found to protect against neuronal cell damage, implicating both cell types in AD pathology (Abramov et al., 2004; Qin et al., 2006). Thus, the plaques themselves lead to the production of toxic ROS and exacerbate both neuronal death and the symptomatic presentation and progression of AD. AD animal models deficient in NOX2 were protected against damage caused by beta-amyloid and did not develop oxidative stress, cerebral dysfunction or behavior deficits of AD (Park et al., 2008). Thus, NOX activation secondary to disease development can exacerbate the disease, providing a potential pathway to mitigate AD progression through NOX inhibition.

Though the etiology of PD is still relatively unclear, oxidative stress and neuroinflammation have been strongly implicated in PD’s loss of dopaminergic cells. This, in part, has been attributed to microglial NOX ROS production and cell damage (Hirsch and Hunot, 2009). Several *in vitro* and *in vivo* models have demonstrated the role of NOX in PD development. In genetic PD *in vitro* and *in vivo* models with knockdown/knockout PINK1, increases in ROS from both mitochondrial and NOX sources were observed, with both the nonspecific NOX inhibitor DPI and NOX-siRNA reducing cytosolic ROS (Gandhi et al., 2009). In *in vitro* rat nigral dopaminergic cells with PD induced by MPTP toxin injections, elevated levels of NOX2 and ROS were observed, both mitigated by NOX inhibition or NOX2 knockdown (Zawada et al., 2011). Similarly NOX1 and ROS were elevated in *in vitro* and *in vivo* PD models induced by 6-OHDA injections, and NOX inhibition reduced cell death (Choi et al., 2012). In PD models in rats, apocynin, a nonspecific NOX inhibitor, was capable of preventing alpha-synuclein aggregation as well as alpha-synuclein mediated NOX activation and ROS production (Sharma et al., 2016). Moreover, NOX inhibition was protective and reduced dopaminergic neuronal death, motor abnormalities, microglial activation and subsequent neuroinflammation after PD was induced via LPS injection into the rat substantia nigra (Sharma and Nehru, 2016; Sharma et al., 2016). Interestingly, a postmortem examination of a small sample of human prePD and PD substantia nigra brain sections suggest that there may be an ANGII/AT1 (ANGII Type 1 receptor)/NOX4 mediated axis of dopaminergic nigral dysfunction. In the brain samples, nuclear AT1 was elevated, though the total AT1 decreased, with increasing disease severity. This accompanied an elevation in NOX4 and caspase-3, suggesting ANGII-activated NOX4 as a possible pathway of dopaminergic cell death in clinical PD (Zawada et al., 2015). Thus, NOX appears to play a key role in both PD development and progression and is a promising therapeutic target, both independently and via upstream signaling pathways such as ANGII.

### Traumatic Brain Injury (TBI)

In TBI, multiple mechanisms cause the final injury, of which oxidative stress and ROS play a pivotal role. NOX acts as a key producer of ROS in TBI, mediating further injury. Though NOX1 to NOX4 are found in the brain, NOX2 increased most after TBI, though an increase in NOX4 was also observed within microglial cells. Moreover, NOX inhibition in TBI models was found to reduce inflammatory injury as well (Cooney et al., 2013). Other models have also demonstrated increases in NOX activity post injury (Lu et al., 2014; Niesman et al., 2014). In TBI models, increased gp91-*phox* (NOX2) was also observed and mice deficient for NOX2 genes were found to have less ROS and oxidative damage, inflammation and secondary damage at the site of injury (Dohi et al., 2010). Moreover, post injury, the brain exhibited NMDA excitotoxicity, which may cause further NOX activation and damage (Niesman et al., 2014). As TBI derives from direct injury and the resulting neuroinflammation responses that occur, the oxidative damage done is likely due to direct ROS damage from NOX rather than through signaling pathways. NOX has emerged as one of the main contributors of ROS in TBI, making it a potent neuroprotective target.

### Stroke

Of CNS disease states, ischemic stroke is one of the most studied conditions for NOX activity. Stroke-induced increases in NOX expression, activity, and ROS-mediated damage have been widely observed post ischemia (Tang et al., 2012; Kochanski et al., 2013; Shen et al., 2016). As part of stroke pathology, the lack of blood flow causes injury to mitochondria to the affected regions of the brain. Once reperfusion occurs, the influx of oxygen cannot be detoxified by the mitochondria. As shown in Figure 1, the influx of excess glucose with reperfusion causes increased production of NADPH via the hexose monophosphate shunt, using the glycolytic intermediate glucose 6-phosphate and producing downstream glycolytic intermediates such as fructose 6-phosphate that go on to enter the tricarboxylic acid (TCA) cycle and produce adenosine triphosphate (ATP) in mitochondria. Thus, with plentiful substrates and a hyperglycemic state, NOX is activated and generates an increased amount of ROS, resulting in subsequent injury. The ROS directly damage cells by compromising membrane integrity via lipid peroxidation and organelle damage. However, they also cause injury by upregulating several inflammatory factors and cytokines, including IL-1β, TNF-α, iNOS, and bradykinin (Chen et al., 2011; Tang et al., 2012). Moreover, bradykinin and IL-1β activate NOX in order to cause blood brain barrier breakdown (Woodfin et al., 2011). NOX2 knockout mice demonstrated reduced inflammation with a reduced elevation of these inflammatory factors, reduced microglial activation and overall reduced infarct size, greatly implicating NOX in both inflammation and infarct progression (Chen et al., 2011).

A hyperglycemic state has been associated with acute stroke and hyperglycemia has independently been found to induce NOX activation as well (Wang et al., 2013; Shao and Bayraktutan, 2014) Hyperglycemic states have been found to potentiate blood-brain barrier breakdown through endothelial apoptosis facilitated by PKC-induced NOX activation of apoptotic pathways and through tight junction dysfunction due to NOX-induced MMP induction (Shao and Bayraktutan, 2013, 2014). Rat models have also shown increased blood-brain barrier permeability and increased infarct and hemorrhage with t-PA reperfusion in hyperglycemic rat models that was reversed with NOX inhibition with apocynin (Won et al., 2011). Thus, hyperglycemia may exacerbate stroke and reperfusion injury.

Moreover, stroke pathogenesis is attributed more to the NOX (NOX2) of circulating immune cells compared to the endogenous microglial and neuronal cells, though both contribute to the stroke pathology (Tang et al., 2011; Kahles and Brandes, 2013). However, a growing body of evidence shows that targeting NOX4 is effective as well, with deletion of NOX4 or targeted inhibition with VAS2870, a NOX4 inhibitor, resulting in reduced oxidative stress, blood brain barrier disruption, neuronal death, and mortality (Kleinschnitz et al., 2010). The prominence of NOX in ischemia/reperfusion pathology is critical to its understanding and to potential treatment options of the disease.

Cerebrovascular conditions potentiate the risk of stroke as well, and these conditions are further mediated by NOX. ROS increases hypertension and atherosclerosis by inducing endothelial dysfunction, vascular remodeling and increased stiffness as well as altering the availability of vasoactive compounds such as nitric oxide to reduce vasodilation capabilities (Williams, 2007; Kinkade et al., 2013). Moreover, NOX is inextricably linked to the inflammatory response, which exacerbates atherosclerosis further (Arenillas, 2015). The isoforms located within blood vessels vary widely, with a widespread presence of NOX4 as well as NOX1, NOX2 and NOX5 in different vessel layers (Takac et al., 2012). However NOX 1 and 2 are the main mediators of vascular damage (Chrissobolis et al., 2012; Takac et al., 2012; Gray and Jandeleit-Dahm, 2015). With such increases in vascular damage, increases in thrombus formation and thromboembolic stroke can increase. Moreover, atherosclerosis and hypertension are associated risk factors and potential causative agents for the disease (Ay et al., 2005; Goldstein et al., 2011). Thus, NOX acts on both the initial cause of stroke and mediates its injurious effects afterwards.

### Amyotrophic Lateral Sclerosis (ALS)

Neurodegeneration seen in ALS has also been attributed largely to oxidative stress. In ALS, neurodegeneration of motor neurons occurs, resulting in muscle weakness, paralysis and death. Oxidative stress markers, including lipid peroxidation products malondialdehyde and 4-hydroxynonenal, were elevated in both patient CSF and serum as well as in animal models of ALS (Simpson et al., 2003). Moreover, in both sporadic ALS patients and animal models, NOX2 was elevated, particularly in microglia in the spinal cord. It was found to mediate protein oxidation, and particularly, modify IGF1/AKT survival pathways in motor neurons to facilitate damage (Wu et al., 2006). In familial ALS, a toxic gain of function mutation in superoxide dismutase 1 (SOD1) is the main cause in 25% of cases. SOD1 regulates NOX by binding *Rac* and inhibiting its GTPase activity and maintaining its active GTP bound form. Normally, it uncouples from *Rac* in the presence of H_2_O_2._ With the mutation, the redox uncoupling is defective and leads to increases in *Rac*/NOX activity and activation as *Rac* remains in its active form (Simpson et al., 2003; Harraz et al., 2008). Moreover, NOX deletion or treatment with apocynin to inhibit NOX prolonged survival and reduced neurodegeneration in mice models and glial cells (Wu et al., 2006; Boillée and Cleveland, 2008; Harraz et al., 2008). Overall, the pathology of ALS implicates NOX-mediated ROS production and damage and provides a novel therapeutic target for the condition.

### NOX-Induced ROS Mechanisms in Cellular Damage

In these disease states, despite normal NOX function acting through protective or biologically necessary mechanisms, the pathological and unbalanced ROS production can damage tissue though multiple pathways. Primarily, it contributes to direct damage. This damage is through direct ROS damage to lipid membranes, causing lipid peroxidation breakdown and potential necrotic death. This can also lead to apoptotic pathway initiation with the release of cytochrome C from the mitochondrial membrane, through mitochondrial damage or upregulation of pro-apoptotic BAX and cause both endothelial and neuronal cell death (Hampton and Orrenius, 1997; Brieger et al., 2012). Necrotic death can also ensue, as seen in ischemia/reperfusion injury. Further damage can be caused through direct damage to DNA itself, further inducing either apoptosis or carcinogenesis if the cell cannot repair itself (Brieger et al., 2012). Moreover, ROS can induce autophagy and at excessive levels cause autophagic cell death, as seen in nerve growth factor deprived sympathetic neurons (Scherz-Shouval and Elazar, 2007).

However, damage is also done through auxiliary signaling pathways. In neuronal damage, cell death results from both direct damage, with ROS and mitochondrial dysfunction, and through the induced inflammation (Lambeth, 2007). ROS can upregulate the production of toxic factors, such as IL-1, TNF-α and more superoxide causing neurodegeneration (Block and Hong, 2005). Several other pathways are involved in a more disease specific manner that allow for disease progression outside of simple cell death, as mentioned in the previous disease states. However, NOX overproduction of ROS seen in pathology can be very cytotoxic through numerous mechanisms.

## Potential Treatments on NOX Activation

NOX inhibition through therapy and targeted drugs has great potential for mitigating disease progression, especially given the widespread involvement of NOX in various disease states. However, targeting NOX specifically is a goal in therapy, given the different roles, particularly in immunity, that NOX has physiologically and the different roles each isoform has in disease. NOX inhibition itself can function through several pathways: (1) To inhibit upstream activators of NOX; (2) To interrupt subunit interactions for activation, preventing assembly; (3) To block the flavocytochrome itself, particularly targeting gp91-*phox* (NOX2) or its catalytic homolog; and (4) To interfere with NOX activity through indirect pathways. However, in many cases, the mechanism of inhibition is unclear or acts on a transcriptional/translational level prior to activation. A general summary of the inhibitors’ location of action is shown in Figure 1.

### A General Inhibition of NOX Action

Several therapeutic interventions are available, include normobaric oxygen (NBO), hyperbaric oxygen (HBO), and hibernation therapy, which inhibit NOX via unspecified mechanisms (Figure 1①). In the cases of NBO and HBO, there is the contradicting thought that increased oxygen would exacerbate NOX-mediated ROS production. However, both NBO and HBO have been found to be paradoxically protective in cases of stroke without contributing to further oxidative stress (Singhal et al., 2002; Schäbitz et al., 2004). In one study, NBO reduced blood brain barrier disruption through reduced levels of NOX2 and NOX-induced MMP-9 while preventing occludin degradation in cerebral ischemia (Liu et al., 2011). HBO also downregulated NOX2 expression and NOX activity and improved functional outcome and neuronal survival due to the decrease in oxidative stress in a subarachnoid hemorrhage model (Ostrowski et al., 2006). Hibernation therapies, such as hypothermia or drug-induced hibernation such as ethanol, are also protective in stroke (Kim and Yenari, 2015; Forreider et al., 2016). Of these, ethanol, by itself and in conjunction with NBO, has been shown to decrease NOX levels and activity in a model of ischemic stroke. It likely acts through an upstream mechanism involving PKC and AKT. It also exhibited reductions in infarct volume, neurological deficit, and ROS levels in a stroke model (Kochanski et al., 2013; Cai et al., 2016). These broad therapies however lack specificity in NOX action and act on multiple levels.

VAS2870 (Vasopharm), 3-benzyl-7-(2-benzoxazolyl)thio-1, 2,3-triazolo[4, 5-*d*]pyrimidine, is another inhibitor and has been found to reduce ROS increases in oxidized LDL stimulated endothelial cells (Stielow et al., 2006). Though its mechanism of action is not certain, recent studies have implicated NOX2 and NOX4. One study demonstrated a reduction in ROS and NOX4 expression in skeletal muscle with VAS2870 and suggested a thiol alkylation and modification mechanism of NOX4 inhibition (Sun et al., 2012). A more recent study with a stroke model suggests it acts by upregulation of microRNA that target NOX2 and NOX4 genes and suppress them in stroke. VAS2870 was also found to reduce infarct volume in stroke as well, providing potential for future use (Liu et al., 2016). While promising, off target effects via thiol alkylation on the ryanodine receptor-Ca^2+^ channel were found, eliminating its activation and potentially causing muscle dysfunction (Sun et al., 2012).

### Inhibition of Upstream Activators of NOX

The first type of inhibitors utilizes upstream targeting of PKC and PKC activation pathways for NOX inhibition as a potential therapy (Figure 1②). PKC inhibitors such as Calphostin C, chelerythrine and ruboxistaurin mesylate (RBX) have all been shown to reduce hypertension. RBX is currently in clinical trials for use in diabetic complications, but it has additional function in NOX inhibition (Williams, 2007). In several studies, high concentrations of PKC inhibitors have reduced ischemic damage *in vivo* or cell death *in vitro*, though more work needs to be done to assess its use for ischemia or neurodegenerative diseases therapeutically (Bright and Mochly-Rosen, 2005). However, PKC inhibitors lack specificity to NOX activation, and PKC is implicated in numerous cellular functions and signaling, creating a potential for varied and severe side effects. Other upstream targets also have shown potential. As previously described, ethanol potentially acts through a PKC/AKT pathway of NOX inhibition (Cai et al., 2016). Total salivanolic acid injection (TSI), an injection derived from Salvia miltiorrhiza, is a therapy targeting a PKC activation pathway. It is currently approved in China for treatment of ischemic stroke, particularly with thrombolytic therapy. Through its use, AMPK expression is enhanced, resulting in the subsequent observed reduction of AKT activation, PKC translocation to the membrane, and overall NOX activation with the failure of migration of the cytosolic subunits to the membrane. It acts via the AMPK/AKT/PKC signaling pathway (Tang et al., 2014). By defining the pathological upstream activators of NOX in specific disease states, NOX inhibition can be facilitated by targeting these upstream players, such as PKC and ANGII.

### NOX Subunit Interactions and their Therapeutic Perspectives

The second type of NOX inhibitors acts via inhibition of NOX assembly (Figure 1③). Understanding NOX activation via p47-*phox* phosphorylation by PKC isoforms and subsequent interaction with the p22-*phox* subunit has opened up new areas of therapeutic investigation. While many of the inhibitors or therapies mentioned previously function by inhibiting upstream NOX activators or through unspecified overall inhibition, greater specificity can be achieved through targeting therapies at subunit interactions.

One of the most promising NOX inhibitors is apocynin, or 4-hydrozy-3-methoxy acetophenone, which blocks migration of p47-*phox* to the membrane and prevents NOX assembly and activation. In ischemic injury, it protected against early lipid peroxidation, neuronal degeneration and death, and glial cell activation as a pretreatment and, at low doses, was found to be effective in improving neurological outcomes, reducing infarct volume and hemorrhage (Wang et al., 2006; Tang et al., 2008). It was also found to reduce glial death and promote survival in ALS mice models (Boillée and Cleveland, 2008; Harraz et al., 2008). However, apocynin acts on NOX1 and 2 more than NOX4, the constitutively active NOX isoform. It also requires myeloperoxidase (MPO) for function as it must dimerize to act on NOX. When acting on NOX4 and in the absence of MPO, apocynin was found to act as an antioxidant scavenger (Heumüller et al., 2008). Thus, its scope of use must be more definitively established.

Another promising drug is gp91-ds-tat, a NOX inhibitor specific to NOX2, and potentially NOX1 (Williams, 2007). It functions through inhibition of p47-*phox* binding to gp91-*phox* through emulating the binding region of gp91-*phox*. As a peptide, it internalizes via a sequence corresponding to the HIV viral coat. In mice with AD, it was shown to reduce ROS as well as cerebrovascular dysfunction that contributes to AD pathology (Park et al., 2008). It potentially has other cerebrovascular benefits, as it has been shown to reduce hypertension in ANGII stimulated models as well (Rey et al., 2001). However, it requires intravenous injection due to limited oral bioavailability as a peptide, restricting its clinical potential (Williams, 2007).

While potential pharmaceuticals and their mechanisms are still being investigated, the uniquely modulated activation of NOX allows for increased specificity to NOX isoforms controlled by cytosolic subunits, primarily NOX1–3. Moreover, understanding the biochemical interactions between subunits opens the field to finding inhibitors that target sequences specific to NOX subunits, lowering the widespread side effects of affecting similar groups on other molecules or upstream activators that are not specific to NOX.

### NOX Flavocytochrome Inhibitors

Certain inhibitors, the third type, target the flavocytochrome itself to inhibit NOX (Figure 1④). Of these, diphenyleneiodonium (DPI) is the most commonly used. It forms an irreversible redox adduct with reduced FAD of  the NOX catalytic core. Thus, it halts superoxide production altogether upon NOX activation through uncompetitive inhibition (O’Donnell et al., 1993). In conjunction with dimethylsulfoxide, a free radical scavenger, it was found to reduce infarct size, blood brain barrier disruption, and neurological damage in a stroke model (Nagel et al., 2007). However, its safety is questionable as other flavoenzymes necessary to metabolism may also be inhibited (O’Donnell et al., 1993; Tang et al., 2012). More recent inhibitors are focusing on generating inhibitors specific to NOX isoforms when targeting the catalytic core. GSK2795039, a small molecular inhibitor, has been recently developed and was found to be protective in paw inflammation and acute pancreatitis mouse models. Unlike DPI, it is specific to NOX2, demonstrating reduction in NOX2 activity via competitive inhibition with little to no inhibition of other NOX isoforms and other flavoenzymes, such as xanthine oxidase or nitric oxide synthase (Hirano et al., 2015). Given the increasing evidence for NOX2’s role in such conditions as stroke and the moderate bioavailability of the drug in the brain, the inhibitor has potential in treating ischemia and neurodegenerative disorders as well (Kahles and Brandes, 2013; Hirano et al., 2015). GKT137831 is another such inhibitor but it is specific to NOX 1/4, allowing for varying specificity and targeting. It has shown potential in cases of fibrotic and inflammatory diseases, including liver fibrosis, pulmonary fibrosis, inflammation, and ischemic retinopathy (Aoyama et al., 2012; Deliyanti and Wilkinson-Berka, 2015). Given its role in inflammation and ischemic retinopathy, it may show potential in cerebrovascular conditions like stroke and in neurodegenerative diseases where neuroinflammation plays a core role in the pathology, but further studies need to be done to investigate its use in neurological diseases. Unlike with NOX2 inhibition, little immunosuppression is observed and no spontaneous pathologies are found upon NOX4 inhibition or deletion (Aoyama et al., 2012). Moreover, it has progressed to clinical trials, showing great promise (Teixeira et al., 2016).

### Indirect NOX Inhibitors

Finally, the fourth type of inhibitors use indirect pathways of action and include angiotensin converting enzyme (ACE) inhibitors and angiotensin receptor blockers, which would function by reducing NOX activation induced by the ANGII/PKC pathway. Statins also inhibit NOX by preventing geranylgeranylation, necessary for *Rac* translocation to NOX (Figure 1⑤ Wassmann et al., 2002; Williams, 2007). They have been shown to reduce ROS production, atherosclerotic lesion size, and even endothelial dysfunction in mild essential hypertension in a small human study (Williams, 2007). Though they have not been studied with regard to neurological diseases, they play an important role through reducing cerebrovascular complications that may ensue from such conditions, including stroke.

## Conclusion and Prospective Studies

NOX plays a detrimental role in a widespread range of diseases through its overproduction of ROS, which then damage tissue directly and initiate deleterious signaling cascades within the body. Thus, NOX inhibition has great potential as a therapeutic treatment that can act across several disease states. The potential for further refinement of NOX inhibition is in understanding the key role of p47-*phox* and its translocation to the membrane subunit p22-*phox* via phosphorylation by PKC. The p47-*phox* subunit SH3 domains are masked at rest, and through serine phosphorylation by PKC isoforms at multiple sites, the polybasic masking domain changes conformation to allow for the SH3 domains of p47-*phox* to interact with the proline rich region of p22-*phox*. It translocates to the membrane to anchor the other cytosolic subunits to the membrane catalytic core and facilitate NOX activation. Through understanding this process, the potential of NOX inhibitors of NOX assembly, function, and upstream activation, as a clinical therapy for neurological, vascular, and metabolic conditions has been unveiled. Particularly, NOX activation can be prevented through the inhibition of subunit assembly by, surprisingly, blocking the interaction between two key subunits, p47-*phox* and p22-*phox*. Further research into finding new potential inhibitors and enhancement of the current ones is still necessary, but investigation into inhibitors of NOX assembly allows for a unique avenue of exploration.

## Author Contributions

YD and RR conceived the study, RR drafted the manuscript, FL made the figure, and all authors, YD, XG, FL and RR critically revised and approved the final manuscript. YD and XG provided research funding.

## Funding

This work was partially supported by American Heart Association Grant-in-Aid (14GRNT20460246; YD), Merit Review Award (I01RX-001964-01) from the US Department of Veterans Affairs Rehabilitation R&D Service (YD), National Natural Science Foundation of China (81501141; XG), and Beijing NOVA program (xx2016061; XG).

## Conflict of Interest Statement

The authors declare that the research was conducted in the absence of any commercial or financial relationships that could be construed as a potential conflict of interest.
